# Effect of Cement Type and Water-to-Cement Ratio on Fresh Properties of Superabsorbent Polymer-Modified Cement Paste

**DOI:** 10.3390/ma16072614

**Published:** 2023-03-25

**Authors:** Hasan Dilbas

**Affiliations:** Civil Engineering Department, Engineering Faculty, Van Yuzuncu Yil University, 65080 Van, Turkey; hasandilbas@yyu.edu.tr

**Keywords:** SAP, cement type, water-to-cement ratio, oxide ratio, rheology

## Abstract

Superabsorbent polymer (SAP) is a material with the ability to absorb liquid and desorb liquid from and to the environment, and it can ensure the internal curing of cementitious composites. Although the fresh state properties of SAP-modified mixtures (SAPCP) are affected and have been investigated nowadays, the rheological properties of SAPCP are still a virgin field and they are worth studying. Hence, the current study was aimed and conducted to observe what occurred if cements with different chemical compositions, various ratios of water/cement (w/c) and SAP were used together. Accordingly, CEM I 42.5R, CEM II/A-LL 42.5R and CEM IV/B (P) 32.5R were selected as binders in the mixtures, and w/c ratios were 0.40 and 0.50 for SAPCPs. In total, 24 mixtures were designed, produced and tested in the laboratory and spreading table tests, Vicate tests, viscosity tests and shear tests were conducted on the fresh state of the mixtures to observe the fresh behavior of SAPCPs. As a result, it was determined that the SAP, cement and w/c combinations considered in the article were effective on SAPCP fresh properties and rheology. However, it was determined that the use of high amounts of SAP in the mixture, high cement fineness and high oxide ratios in the cement (ratios of silicon dioxide/calcium oxide and aluminum oxide/calcium oxide) negatively affected not only the fresh state properties, but also the rheology. Moreover, the coexistence of the aforementioned negative conditions was the most unfavorable situation: high SAP ratio + high cement fineness + high oxide ratio in SAPCP. For these reasons, it was concluded that cement fineness and chemical composition should be taken into account in the rheology/workability-based design of SAPCPs. Then, the SAP content can be regulated for design purposes.

## 1. Introduction

With their absorption ability, superabsorbent polymers (SAPs) affect the fresh properties (i.e., workability and rheology) of cement paste (CP) with/without inclusions, such as mineral addition, chemical admixtures, etc. A SAP is able to absorb water many times its own weight, and the desorption and absorption kinetics of SAPs play a vital role in the workability and rheological properties of SAP-modified cement paste (SAPCP). The chemical characteristics, structure and particle dispersion are some factors affecting the de- and absorption behaviors of a SAP, and it was reported that the rheological parameters of SAPCPs, such as viscosity and shear stress, were controlled by the kinetics of the SAP [1]. In addition, the hydration of the CP utilizes free water in the SAPCP, leading to a decrease in workability and a negative impact on the rheological parameters. Accordingly, it seems possible that both the ab-/desorption kinetics of SAPs and the hydration of CPs have an impact on the fresh properties of SAPCPs.

To date, many researchers have tried to increase the workability by adding extra water to increase the overall water content of the mixture, and the water-to-cement proportion (w/c) was then spoiled and increased. Thus, although the required fresh properties of CP were obtained, the target strength value (i.e., compressive strength) and durability properties were not achieved [1]. On the other hand, in some cases, some chemicals were employed to overcome low workability problems due to the consideration of SAP in the paste recovering the target slump value when the mixing water amount was kept constant [1]. Senff et al. conducted a research program that investigated extensive parameters (water-to-cement ratio (w/c), SAP proportion and plasticizer dosage) using a flow table spread value and a rheometer (Viskomat PC) for comparison purposes and concluded that the yield stress of CP significantly depended on CP composition and time [2]. Ma et al. employed a rotational rheometer to obtain rheological parameters such as yield stress, viscosity, thixotropy of SAPCP, etc. over a duration of 80 min, and dry acrylic acid-based acrylamide with various gradings was used in CPs with w/c ratios starting from 0.18 up to 0.24. It was concluded that the particle size of the SAP influenced the speed and amount of absorbed water content [3]. It can be noted that the low w/c ratio (<0.35) decreased the workability and increased the strength, but a 0.45 w/c ratio was effective for gaining strength/durability and maintaining enough workability. Hence, the values of w/c were selected as 0.40 and 0.50 in the current paper. In addition, Mechtcherine et al. [4] used Portland cement and SAPs with various sorption characteristics and examined the rheological parameters of CPs. It was stated that larger particles of SAP increased their weight by the absorption of water throughout the experimentation time [4].

However, the water absorption of SAPs sometimes became limited due to the presence of finer mineral particles (i.e., silica fumes) [4]. On the other hand, temperature was as effective of a factor in the rheology of CP as water content, and Secrieru et al. [5] investigated the effect of temperature on the rheological properties of SAP-modified cement-based composites. It was reported that SAPs positively influenced the yield stress and plastic viscosity independent of temperature. On the other hand, the hardened states of SAPCPs were investigated by some researchers, who determined the impact of SAPs on physical, mechanical and durability properties; the most notable work in the current literature is RILEM’s penned report on SAP effects [1]. In the report, it was stated that the use of SAP reduced the drying shrinkage of the mixtures, decreased the strength, improved the durability properties and increased the resistance to freeze-thaw cycles and high temperature. In addition, it has been found that the strength of the samples containing SAPs can be increased by curing at temperatures up to 100 °C. However, it was observed that the relationship between cement type and hardened properties did not work very well.

Although these papers considered the effects of SAP properties, mixture design parameters (w/c ratio), environmental impact (i.e., humidity and temperature), etc. on the fresh state properties of SAPCPs, it was observed that the main parameters of mixtures (cement, etc.) and their impacts on fresh state properties were rarely studied. In this experimental study, three types of cement were employed to investigate the impact of the cement type and its chemical composition on the rheological parameters/fresh properties. A total of 24 SAPCPs were composed. Then, tests such as spreading, setting start time/setting end time, viscosity and shear stress value of the SAPCP were properly applied to the fresh mixtures. Then, the results were evaluated.

## 2. Materials

Three types of cement (Portland Cement (CEM I 42.5R, 90% clinker + 5% gypsum + 5% limestone), Portland Calcareous Cement (CEM II/A-LL 42.5R, 75% clinker + 5% gypsum + 20% limestone) and Pozzolanic cement (CEM IV/B (P) 32.5R, 50% clinker + 5% gypsum + 5% limestone + 40% pozzolan)) were used in the mixtures, and their chemical contents are given in Table 1. The water used for mixing was pure and contained no minerals. A commercially available SAP (Tusorb JK-01, Mekitem Medikal Kimya Trade Company, Istanbul, Turkey) was employed in the mixes and was based on a cross-linked copolymer of acrylamide. It absorbs water up to 60–150 times its volume in 2–5 min. The properties of the SAP are given in Table 1. 

## 3. Method

### 3.1. Mix Proportions

A total of 24 mixes were designed to assess the effect of the cement type, the cement chemical composition and the water-to-cement ratio on the fresh state properties of SAPCP. The components of the mixes are given in Table 2. Three types of cement and two types of water-to-cement ratios: 0.40 and 0.50, were chosen as variables in the mix design of SAPCP. The mix volume was selected to fill the volume of the Brookfield Rheometer device apparatus.

### 3.2. Mix Procedure

After the weighing process, the dry components of the SAPCP (cement and SAP) were added into a container, and pure water was added into the container. Then, the dry mix was wetted with enough water according to the composition details of the SAPCP and was mixed by hand. When the workability of the mix became constant, the mix was tested.

### 3.3. Testing

#### 3.3.1. Flowability

A flow table test was applied to mixes to observe the consistency of the fresh state of each SAPCP. The flow value of SAPCP was assessed in consideration of a mini-slump test with a cone having 70 mm and 100 mm top and bottom diameters, respectively. According to the test, first, the conic mold was filled with fresh mortar. Then, the mold was pulled up vertically and the mortar flowed. The flow table was vibrated at a constant frequency. In the end, the average of the two perpendicular dimensions of the SAPCP was noted and the average of the dimensions was recorded as the final diameter.

#### 3.3.2. Stiffening

To determine the early stiffening of each SAPCP, a Vicate device was employed in the experiments. SAPCPs were prepared with different w/c ratios, cement types, and SAP proportions. After the wet mixing of the materials, the Vicate test was performed on each fresh SAPCP. The testing times were chosen as 0, 10, 20, 30, 40, 50 and 60 min, and higher values of time were not considered because this paper considers the fresh properties of SAPCPs after wet mixing. The long-term rheology of SAPCP will be evaluated in further research.

#### 3.3.3. Rheology

**Figure 1:**
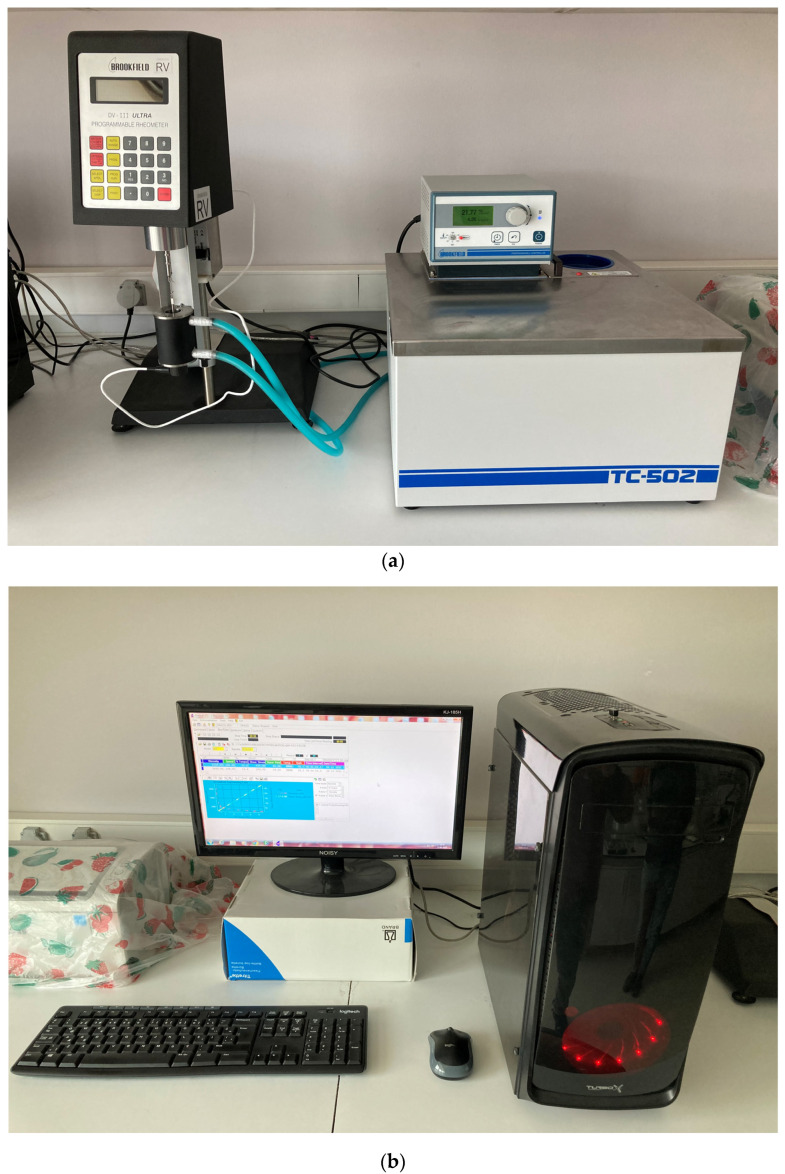

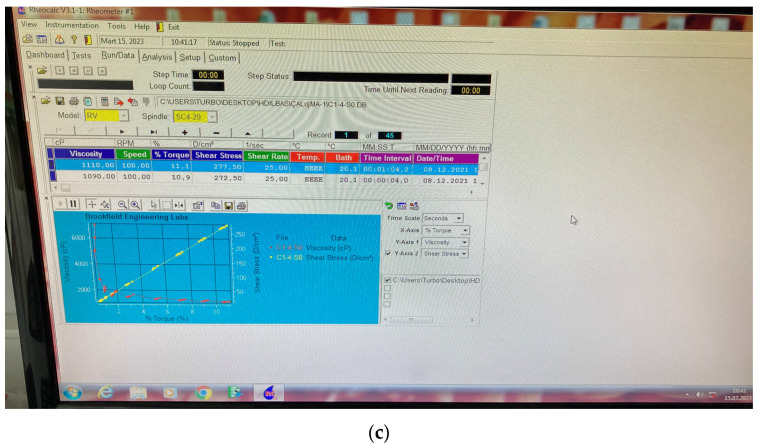
Test setup and details: (**a**) the rheology test, temperature regulator and testing device part with spindle and sample container; (**b**,**c**) data evaluation was obtained with device software.

The mixing procedure was completed for all SAPCP mixes, and a Brookfield RV device with an SC4-29 spindle was employed in the tests (Figure 1). The developed procedure started with a pre-shearing cycle for a certain time (60 s). Then, different shear rates were applied for a certain time to observe the fresh state response of each SAPCP (Table 3). It is notable that, at first, the mixing procedure and time accuracy for each step were tried on trial mixes, and then the tests were begun after the positive results of the trials.

## 4. Results

### 4.1. Flowability and Initial/Final Setting Time

The results of the spreading table and Vicate tests are given in Table 4. When the results of SAPCPs containing CEM I 42.5R were examined, it was determined that the spreading values were obtained as 21, 19, 17 and 14 cm, respectively, in SAPCPs containing 0.40 w/c and 0–0.25–0.50–0.75% SAP. It was observed that the SAP additive decreased the spreading values, and the minimum spreading value was obtained as a result of using 0.75% SAP. In the case of the same series containing 0.50 w/c and 0–0.25–0.50–0.75% SAP, it was determined that the spreading values were 25, 23, 22 and 20 cm, respectively, and the SAP content decreased the spreading values, as shown. It was seen that these values were larger than the series containing 0.40 w/c. On the other hand, when the results of SAPCPs containing CEM II/A-LL 42.5R were examined, the spreading values were obtained as 19, 18, 17 and 15 cm in the series containing 0.40 w/c and 0–0.25–0.50–0.75% SAP, respectively. For the same series containing 0.50 w/c and 0–0.25–0.50–0.75% SAP, the spreading values were determined to be 20, 19, 18, and 16 cm, respectively. It was seen that the SAP content reduced the spread values, but these values were greater than the series containing 0.40 w/c. It was determined that this situation was similar to the series containing CEM I 42.5R and that the SAP additive reduced the spreading values. In the case of both cement types (CEM I 42.5R and CEM II/A-LL 42.5R), it was seen that the minimum spreading value was obtained as a result of using 0.75%. In addition, the results of the third cement type (CEM IV/B (P) 32.5R) showed that within the scope of the study with varying amounts of SAP, increasing SAP content decreased the spreading values. However, it was seen that this reduction value was higher than those shown in the cases of using the other two cements (CEM I 42.5R and CEM II/A-LL 42.5R). From this point of view, it was determined that the effect of spreading values with the change of cement type was able to be clearly observed. It was thought that the fineness and chemical composition of the cements used had clear effects on the results, and details are given below. Additionally, here it was observed that the thinness values of CEM I 42.5R, CEM II/A-LL 42.5R and CEM IV/B (P) 32.5R were 3871, 4655 and 4358 cm^2^/g, respectively, and CEM II/A-LL 42.5R and CEM IV/B (P) 32.5R fineness values seemed to be higher than CEM I 42.5R fineness values. It was thought that as the fineness value increased, the water value that the surface could hold increased with the increase in surface area, and the increase in cohesion accelerated the solidification by absorbing the water in the environment [1]. On the other hand, although they had approximately the same fineness, it was determined that the spreading values obtained as a result of the use of CEM II/A-LL 42.5R and CEM IV/B (P) 32.5R cements in the mixtures were quite high. It was commented that the chemical composition of the series containing CEM IV/B (P) 32.5R may have been an effective factor, in addition to the fineness value, in the lower spread value. As given in Table 1, while CEM IV/B (P) 32.5R contained relatively more silicon oxide and aluminum oxide, CEM II/A-LL 42.5R contained relatively more calcium oxide (other values were approximately the same). It was thought that this difference caused an effect in the spreading values, and it was noted that it would be appropriate to examine the effect of the ratio of silicon oxide and aluminum oxide to calcium oxide on the fresh state properties of SAPCPs. Hence, the ratios of oxides were used in the evaluation of the results, not only in this section, but also in other sections. When the ratios of silicon oxide/calcium oxide and aluminum oxide/calcium oxide were calculated for CEM I 42.5R, CEM II/A-LL 42.5R and CEM IV/B (P) 32.5R, silicon oxide/calcium oxide and aluminum oxide/calcium oxide ratios were 0.31 and 0.09 for CEM I 42.5R, 0.31 and 0.08 for CEM II/A-LL 42.5R and 0.92 and 0.23 for CEM IV/B (P), respectively. Thus, it was found that the higher ratios corresponded with lower flowability. When the yield stress values in this view were analyzed with the obtained ratios, it was seen that CEM IV/B (P) 32.5R stood out by far. The detailed evaluation is given in the following sections. The effect of the SAP’s water absorption was evident in the spreading and setting time properties of SAPCPs. On the other hand, the decrease in the free water content of the mixture due to SAP absorption reduced the setting times; although the use of 0.75% SAP in the SAPCPs had a significant effect, the time between start and finish times was approximately the same. 

### 4.2. Rheology

**Figure 2:**
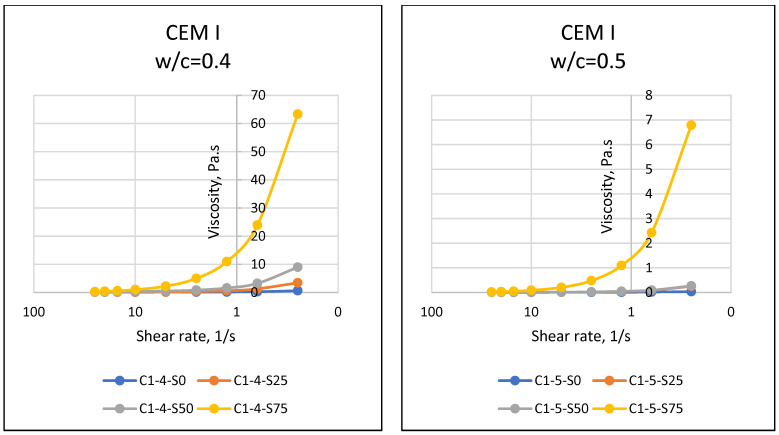
Viscosity–Shear Rate Relationships of SAPCPs with CEM I (w/c = 0.40 and 0.50).

**Figure 3:**
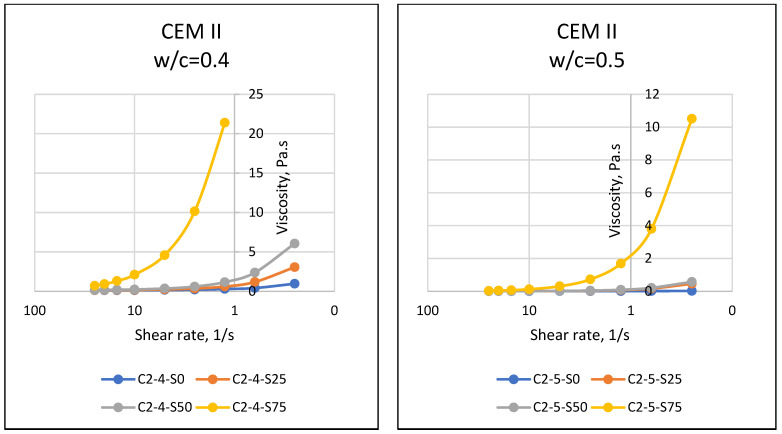
Viscosity–Shear Rate Relationships of SAPCPs with CEM II (w/c = 0.40 and 0.50).

**Figure 4:**
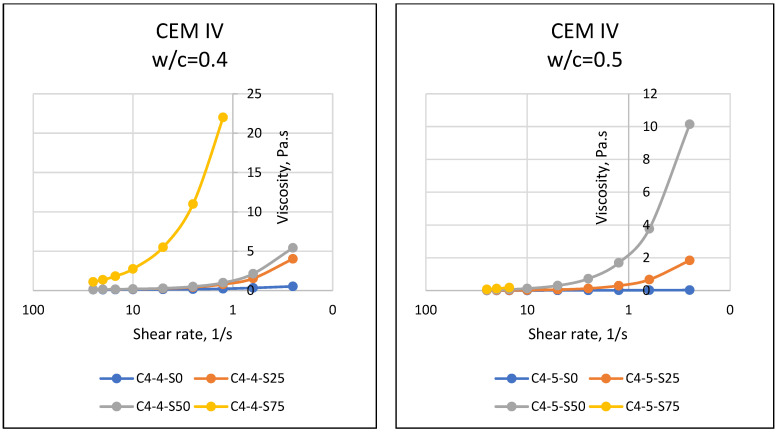
Viscosity–Shear Rate Relationships of SAPCPs with CEM IV (w/c = 0.40 and 0.50).

**Figure 5:**
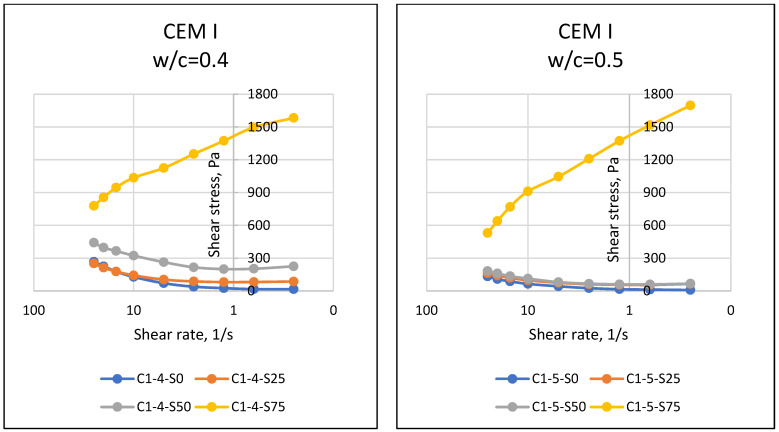
Shear Stress–Shear Rate Relationships of SAPCPs with CEM I (w/c = 0.40 and 0.50).

**Figure 6:**
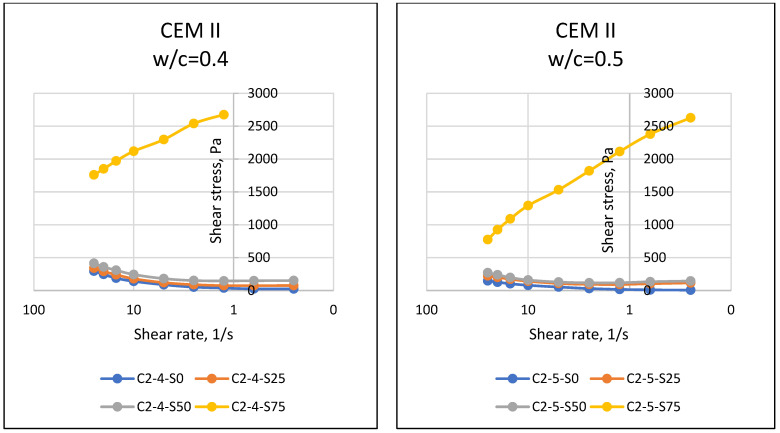
Shear Stress–Shear Rate Relationships of SAPCPs with CEM II (w/c = 0.40 and 0.50).

**Figure 7:**
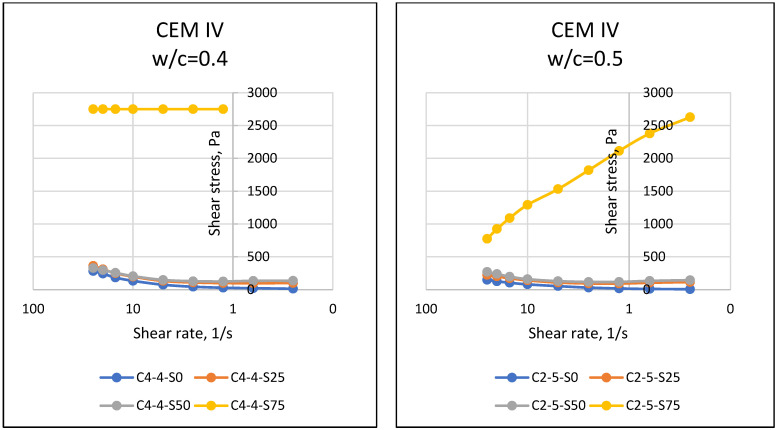
Shear Stress–Shear Rate Relationships of SAPCPs with CEM IV (w/c = 0.40 and 0.50).

If matter, especially fluids, is forced to move towards the center of the earth with the effect of gravity, the resistance of the substance against this motion reveals the dual force balance, and sometimes the imbalance. At this point, the motion instability of the object results in flow, and the resistance of the object against flow emerges as a property of the object. The resistance to flow mentioned in rheology (flow science) is defined as the viscosity of the substance. In general, it is known that while it is possible for a substance to flow easily and gain movement at relatively low viscosity values, substances that do not flow easily have difficulty in flowing due to their relatively higher viscosity. Viscosity properties can come to the forefront in matters such as easy flow of cement-bound materials, thus affecting the easy settling of the material and its ability to take the shape of the mold. In this respect, the viscosity parameter, which is one of the rheological properties of materials, should be known and controlled. Considering the properties of the materials used in this article, it was expected that the particles of cement with fine grains would retain water with a cohesive effect, and SAP, which has a high water absorption capacity, would absorb the water in the environment. In addition, different chemical properties of cement may have an effect on the rheological properties of the mixture, and it was necessary to accurately determine the fresh state properties and rheological behavior of SAPCPs. The viscosities of the SAPCPs are presented in Figure 2, Figure 3 and Figure 4. The figures show that the viscosities of the SAPCPs increased due to the reduction in shear rate. In addition, the previously observed and mentioned increase indicated a nonlinear relationship between viscosity and shear rate. It was stated that the water content played an important role in the viscosity: the higher w/c ratio, the lower the viscosity, regardless of the cement type. Comments on the evaluation of the viscosity–shear rate relationship are given below according to the cement types used in the tests. In addition, the shear stresses of SAPCPs are given in Figure 5, Figure 6 and Figure 7, and it was found that the shear stress–shear ratio relationship was not linear.

#### 4.2.1. Viscosity

The results given in Figure 2 show a nonlinear inverse relationship between the viscosity and shear rate of CEM I 42.5R and SAPCPs at 0.40 and 0.50 w/c. The viscosity of the SAPCP increased due to the rapid absorption of the free water of the mixture by the SAP, and the increase in viscosity of SAPCPs with 0.40 w/c was higher than 0.50 w/c. Since the free water per cement was higher for SAPCPs with w/c = 0.50 than for SAPCPs with w/c = 0.40, the difference in ratios resulted in higher workability and therefore lower viscosity. The results given in Figure 3 show that there was an inverse nonlinear relationship between the viscosity and shear rate of SAPCPs containing CEM II/A-LL 42.5R and w/c = 0.40 and 0.50, as with CEM I 42.5R. The free water of the mixture was rapidly absorbed by the SAP and the viscosity drop of SAPCPs containing w/c = 0.50 was lower than those containing w/c = 0.40. It was thought that the water content of the SAPCP may be a factor in the mentioned behavior, resulting in a lower viscosity due to the higher water content. However, when the viscosities of SAPCPs containing CEM II/A-LL 42.5R were compared with that of CEM I 42.5R, the viscosities of SAPCPs containing CEM II/A-LL 42.5R (for w/c = 0.40) were lower than SAPCPs containing CEM I 42.5R. CEM I 42.5R and CEM II/A-LL 42.5R had approximately the same chemical compositions, but Blaine values were thought to cause the differences in the results. Since smaller particles can absorb more of the free water content of the mixture, it causes not only a decrease in the workability of the SAPCP, but also an increase in its viscosity [1,3,6,7]. The results given in Figure 4 show that there was an inverse nonlinear relationship between the viscosity and shear rate of SAPCPs with CEM IV/B(P) 32.5R for w/c = 0.40 and 0.50. This situation was similar to the SAPCPs described above. The viscosity of the SAPCP increased due to the absorption of the water of the mixture by the SAP. The increase in viscosity of SAPCPs with w/c =0.40 was higher than that of SAPCPs with w/c = 0.50. It is possible to comment that the free water content of the mixture was a factor in the low viscosity. However, when the viscosities of SAPCPs containing CEM IV/B (P) 32.5R were compared with the viscosities of SAPCPs containing CEM I 42.5R and CEM II/A-LL 42.5R, the viscosities of SAPCPs containing CEM IV/B (P) 32.5R (w/c = 0.40) were lower than those of CEM I 42.5R, and similar to CEM II/A-LL 42.5R. It is thought that the similar Blaine values of CEM II/A-LL 42.5R and CEM IV/B (P) 32.5R caused similar results, while different Blaine values of CEM I 42.5R and CEM IV/B (P) 32.5R caused different viscosity results. In addition, when the viscosities of SAPCPs including CEM II/A-LL 42.5R and CEM IV/B (P) 32.5R were compared, the viscosity of SAPCPs containing CEM IV/B (P) 32.5R was found to be different from the viscosity of SAPCPs containing CEM II (w/c = 0.50). It was thought that the mentioned issue may be caused by the chemical composition of CEM II/A-LL 42.5R and CEM IV/B (P) 32.5R. The other properties of CEM II/A-LL 42.5R and CEM IV/B (P) 32.5R were approximately the same, except for their chemical content. However, it was seen that the silicon oxide and aluminum oxide contents of CEM II/A-LL 42.5R and CEM IV/B (P) 32.5R were different, while iron oxide contents of the cements were about the same. It was reported in the literature that aluminum oxide and iron oxide showed faster hydration behavior than other components of cement, accelerated the hardening of the cement and reduced the workability [8,9]. On the other hand, silicon dioxide was thought to have a similar effect. In addition, the high silicon oxide/calcium oxide ratio and aluminum oxide/calcium oxide ratio of CEM IV/B (P) 32.5R may have had an effect on the test results. When the chemical properties of CEM I 42.5R type cement were examined, it was seen that the ratios were low compared to the others. Thus, there was a possibility that this may have lead to an increase in the viscosity of the SAPCPs. In addition, high SAP content in the mixture had an effect on the high viscosity of the SAPCP. The use of high SAP content in the mixture and CEM IV/B (P) 32.5R with high alumina content may have had a dual effect. This prevented the accurate measurement of the viscosity of the SAPCP and caused rapid hardening of the SAPCP, although the SAPCP with w/c = 0.50 had higher viscosity than the SAPCP with the water content at w/c = 0.40. It was considered that the chemical composition of the cements was different and influenced the spreading test results as stated above. When the viscosity results were evaluated regarding the relationship between viscosity and the ratios of silicon dioxide/calcium oxide and aluminum oxide/calcium oxide, the viscosity of the SAPCPs including the three cements were interesting. For instance, regarding 0.75% SAP use in SAPCPs, although the viscosity values could be assessed for the SAPCPs including CEM I 42.5R and CEM II/A-LL 42.5R, it could not be measured for SAPCPs with CEM IV/B (P) 32.5R. When the ratios of silicon oxide/calcium oxide and aluminum oxide/calcium oxide of cements such as CEM I 42.5R, CEM II/A-LL 42.5R and CEM IV/B (P) 32.5R were evaluated, the highest ratios were seen in CEM IV/B (P) 32.5R. It was noted that the higher the oxide ratios, the higher the viscosity.

#### 4.2.2. Shear Stress

Figure 5, Figure 6 and Figure 7 present the relationship between shear stress and shear velocity, taking into account various SAP ratios of 0–0.25–0.50–0.75%. Accordingly, an increase in shear rate caused fluctuation in the shear stress of SAPCPs. The observed behaviors are called shear thinning and thickening behaviors, for an increase and decrease in shear stress of SAPCPs, respectively, in rheology terminology. Both shear thinning and thickening behaviors were determined as nonlinear behaviors, and only shear thinning behavior was determined for SAPCPs with 0.75% SAP. Except for the use of 0.75% SAP in the SAPCP mixtures, it was seen that the increase in shear rate decreased the viscosity and increased the shear stress. The consideration of cement types in SAPCP mixtures, and thus the evaluation of the relationship between shear stress and shear rates are discussed separately below. When Figure 5 was examined, it was found that there was a non-linear relationship between shear stress and shear velocity, except for mixtures containing 0.75% SAP. Due to the increase in internal friction of SAPCP, the shear stress value increased. It has been interpreted that the shear stresses of SAPCPs with a w/c ratio of 0.40 and 0.50 were caused by the free water content, which reduced the internal resistance/friction, because the water content in the SAPCP mixture reduced the internal particle friction by providing lubricity between the particles. In addition, the increase in the SAP content in the mixture absorbed the free water at a high rate, and this situation caused the loss of free water, which affected the internal friction and increased the loss of workability. Figure 6 shows the results of the shear stress–shear velocity relationships for SAPCP blends containing CEM II/A-LL 42.5R. When the results were compared, it was clear that there was a similarity between the CEM I 42.5R and CEM II/A-LL 42.5R results for each SAP content. It was determined that the increase in the SAP content of the mixtures increased the shear stress value of the SAPCP, and the shear stress values of the SAPCPs containing 0.40 w/c were greater than those containing 0.50. On the other hand, when the shear stress–shear velocity results of SAPCPs containing CEM I 42.5R and CEM II/A-LL 42.5R were compared, the shear stress of SAPCPs containing CEM II/A-LL 42.5R was found to be higher than those containing CEM I 42.5R. The chemical compositions of CEM I 42.5R and CEM II/A-LL 42.5R were approximately the same, while the fineness values of these cements were different, and it was thought that the shear stress values were affected due to the Blaine fineness. Therefore, it was interpreted that the rheological properties were affected because the fine particles had more surface area than the coarse particles; it has been observed that the free water in the environment was absorbed by the fine particles due to cohesion and the workability of the mixture decreased, increasing the internal friction and shear stress [1,3,10]. On the other hand, the results of mixtures containing CEM IV/B (P) 32.5R were as shown in Figure 7. The results show that the high fineness of CEM IV/B (P) 32.5R and the high SAP content in the SAPCPs caused rapid absorption of free water in the environment and increased shear stress with reduced workability.

#### 4.2.3. Yield Stress

**Figure 8:**
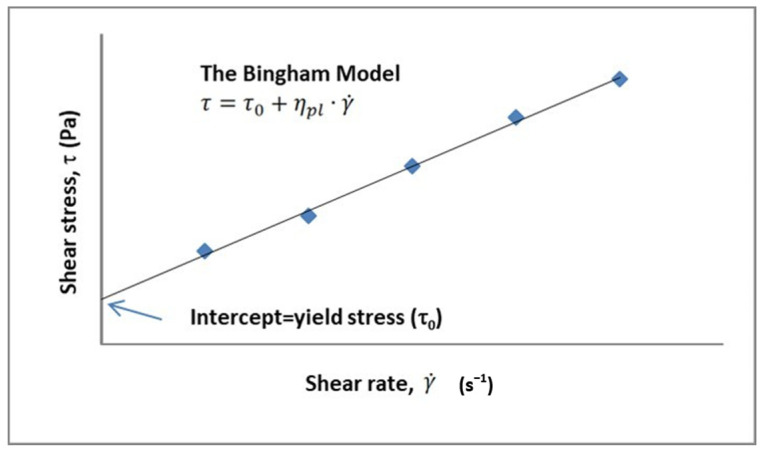
Bingham model [11].

**Figure 9:**
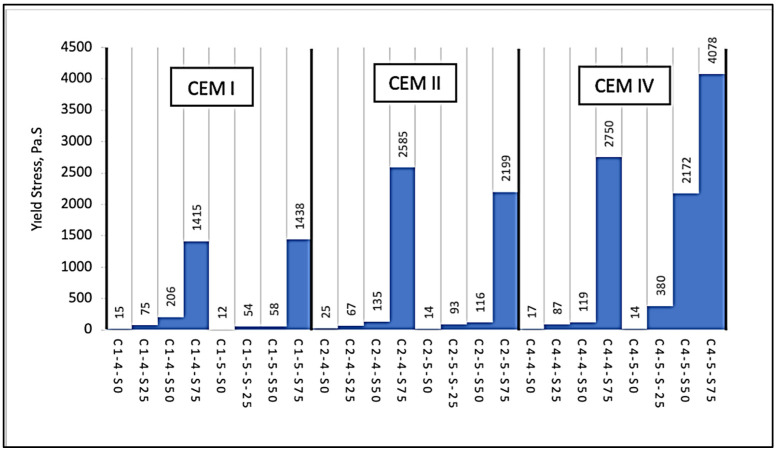
Yield stress values of SAPCPs, including different cement types and SAP contents.

The various ingredients/materials used in the mixtures were the main factors that affected the behavior of the mixtures. The interactions between the components were encountered as a matter that needed to be examined secondarily. The rheology of the mixture was a fresh state material parameter that was formed and changed with the environmental conditions under these two effects. Through the physical and chemical interactions of the solids in the solution with each other and with the liquid medium, the yield stress was formed from the rheological parameters of the mixture. By definition, the yield stress was seen as a line in the solid-liquid behavior of the solution. A solution that can pass the yield stress can flow, and this threshold is especially important in systems with liquid media containing solid particles. In this study, the SAPCPs contained water as the liquid, and the solid particles were the cements (here, CEM I 42.5R, CEM II/A-LL 42.5R and CEM IV/B (P) 32.5R) and SAP grains in varying proportions. Different types of cements examined as the subject of this study have different compositions and grain sizes, and details such as fineness values and SAP properties were presented in Section 2 “Materials”. Considering the aforementioned features, the water content added for the hydration and workability of the cement paste, which was formed by the SAP, a material with a high water absorption capability, and different types of cements with relatively high fineness (3800> cm^2^/g), would be absorbed by both the SAP and the cement grain. Due to the lack of water in the environment, the mixture looked like a liquid at first, but then behaved as a solid. At this point, the rheological properties examined in this article aim to shed light on cases of possible SAPCP use for researchers and end users. The yield stress expressed the magnitude of the stress value required for the mixture to start flowing. As is known, it was important for the mixture to be fluid and to be able to take the shape of the container it was in by flowing easily, both in terms of workability and final strength [8]. Using the obtained rheological test results, the data fitting process regarding the test results was made utilizing the Bingham Model detailed in Figure 8. After the evaluation, it was concluded that the results were compatible with the Bingham Model and the correlation coefficient was found as R > 0.95 (which indicates high significance/accuracy) (Table 5). The results show that the SAP content in the cement types predominantly affected the yield stress of the SAPCPs (Figure 9). As expected, the results ranged from approximately zero to infinity due to the increased amount of SAP in the blends. However, with different cement Blaine values and chemical compositions, cement type ranked as the second most impactful factor after SAP content.

When the results of the series containing CEM I 42.5R were examined, it was seen that the yield stress value increased from 15.1 Pa to 1414.8 Pa when SAP was used at increasing rates from 0% to 0.75% in SAPCPs containing 0.40 water/binder. In the case of 0.5 water/binder use, it increased from 11.9 Pa to 1438.3 Pa. In the case of using CEM I 42.5R, it was determined that increasing the water/binder ratio reduced the yield stress. However, if the highest utilization rate of 0.75% SAP was used, it has been observed that the yield stress—despite the increased water/binder ratio—was approximately the same as the SAPCP containing 0.40 water/binder. In the mixtures containing CEM II/A-LL 42.5R, the behavior was similar to that of the mixtures containing CEM I 42.5R, but higher yield stress values were obtained for each SAP usage ratio in the SAPCPs containing CEM I 42.5R. For example, the yield value of SAPCP with 0.50 w/c ratio, 0.75% SAP, and CEM I 42.5R was determined as 1438.3 Pa. If the cement type was chosen as CEM II/A-LL 42.5R at the same mixing ratios, the yield stress value was obtained as 2199.0 Pa. It was thought that this difference in the results obtained—that is, the increase in yield stress—may be due to the fineness of the cement (CEM II/A-LL 42.5R had a Blaine fineness of 4655 cm^2^/g, while CEM I 42.5R had a fineness of 42.5R 3871 cm^2^/g). Considering that the chemical content of these two types of cement was almost the same, but the fineness differed, it could be argued that the fineness of the cement also had an effect on the rheological parameters, in addition to the use of SAP. When the results of the series containing CEM IV 32.5R were examined, it was seen that the yield stress values increased with increasing the SAP content in the SAPCP. When the series containing CEM IV 32.5R and CEM II/A-LL 42.5R were compared, it was observed that the cements in the mixtures had approximately the same fineness values but had different chemical contents. It was seen that the silicon dioxide and alumina content of CEM IV 32.5R was relatively higher, while the calcium oxide content of CEM II/A-LL 42.5R was higher. It was thought that this difference may have had an effect on the yield stress, and it can be stated that the yield stress values were increased by oxide content. When 0.75% SAP content and 0.50 water/binder ratio were considered, SAPCPs with CEM IV 32.5R and CEM II/A-LL 42.5R had yield stress values of 4077.5 Pa and 2199.0 Pa, respectively. When SAPCPs containing CEM I 42.5R and CEM IV/B (P) 32.5R cements were compared, considering both the cement fineness and the difference in cement chemical properties, the series containing CEM IV/B (P) 32.5R had higher yield stress. On the other hand, when the ratios of silicon oxide/calcium oxide and aluminum oxide/calcium oxide of cements were considered, the higher ratios were found in CEM IV/B (P) 32.5R, with values of 0.92 and 0.23, respectively, and the higher yield stresses were usually found for SAPCPs with CEM IV/B (P) 32.5R. It can be said that the differences due to cement type was observed clearly and the relations between the oxide ratios and fresh properties of SAPCP were evaluated.

## 5. Conclusions

In this article, the effect of superabsorbent polymer (SAP) content, water/cement ratio (w/c) and cement type on the fresh properties of SAP-modified cement paste (SAPCP) was investigated. Accordingly, CEM I 42.5R, CEM II/A-LL 42.5R and CEM IV/B (P) 32.5R; 0.40 and 0.50 w/c; and 0.25, 0.50 and 0.75% SAP were the components/design parameters of the SAPCP mixtures. The following remarks are stated as conclusions:

- Effect of SAP: The use of SAP in SAPCP blends worsened the fresh state properties of the blends due to the SAP absorbing water from the environment. For this reason, as a result of the use of SAP, the spreading table value and initial/final setting time decreased, and the viscosity value, the shear stress value and the yield stress value increased. As a result of the use of increasing amounts of SAP in SAPCP mixtures, the described negative situations became worse.
- Effect of water-to-cement ratio: The amount of water used in SAPCP mixtures affects the flow properties of the mixtures; increasing the w/c ratio resulted in increased spreading table value and initial/final setting time, and decreased viscosity value, shear stress value and yield stress value.
- Effect of cement type: The three cement types considered in this study have different chemical contents and different Blaine fineness values. As the fineness of the cements increased, the surface area of the cement grains increased. As a result, the water in the environment was absorbed more effectively by the cement grains and the fresh state properties of SAPCP were adversely affected. In addition, the effects of different chemical contents (especially SiO_2_, Al_2_O_3_ and CaO) in the chemical structure of cements on the fresh state were determined. Considering the oxide ratio values, it was determined that CEM IV cement had the highest SiO_2_/CaO and Al_2_O_3_/CaO ratios, and it was observed that the most unfavorable fresh state properties were often in the mixtures with the highest ratios. It has been suggested that a mixture design can be made by taking into account the oxide ratios and cement fineness.

## Figures and Tables

**Table 1:** Cement’s and SAP’s properties.

Cement Type	SiO_2_	Al_2_O_3_	Fe_2_O_3_	CaO	MgO	SO_3_	K_2_O	Na_2_O	Loss
CEM IV/B (P) 32.5R	33.98	8.44	3.59	36.74	1.77	2.48	2.09	0.64	5.47
CEM II/A-LL 42.5R	18.79	4.87	3.15	60.63	2.64	2.95	0.76	0.34	10.47
CEM I 42.5R	19.19	5.25	3.21	61.52	2.79	2.70	0.82	0.35	3.37
	Blaine, cm^2^/g	Cum. passing sieve 120 μm		Cum. passing sieve 90 μm		Cum. passing sieve 45 μm		Cum. passing sieve 32 μm	
CEM IV/B (P) 32.5R	4358	100		98.56		45.18		6.96	
CEM II/A-LL 42.5R	4655	100		97.23		44.63		8.07	
CEM I 42.5R	3871	100		95.21		33.65		6.15	
SAP	-	61.43		18.11		7.65		1.56	

**Table 2:** Mix components for each series.

Notation	Water-to-Cement Ratio	Cement Type	Cement, g	Water, g	SAP, g
C1-4-S-0	0.40	CEM I	30	12	0
C1-4-S-25			30	12	0.075
C1-4-S-50			30	12	0.150
C1-4-S-75			30	12	0.225
C1-5-S-0	0.50		30	15	0
C1-5-S-25			30	15	0.075
C1-5-S-50			30	15	0.150
C1-5-S-75			30	15	0.225
C2-4-S-0	0.40	CEM II	30	12	0
C2-4-S-25			30	12	0.075
C2-4-S-50			30	12	0.150
C2-4-S-75			30	12	0.225
C2-5-S-0	0.50		30	15	0
C2-5-S-25			30	15	0.075
C2-5-S-50			30	15	0.150
C2-5-S-75			30	15	0.225
C4-4-S-0	0.40	CEM IV	30	12	0
C4-4-S-25			30	12	0.075
C4-4-S-50			30	12	0.150
C4-4-S-75			30	12	0.225
C4-5-S-0	0.50		30	15	0
C4-5-S-25			30	15	0.075
C4-5-S-50			30	15	0.150
C4-5-S-75			30	15	0.225

**Table 3:** Testing procedure.

**Shear rate, rpm**	100 *	100	80	60	40	20	10	5	2.5	1
**Time, second**	60	15	15	15	15	15	15	15	15	15

* Pre-shearing.

**Table 4:** Test results of flow table and Vicate testing.

Notation	Flow Diameter, (cm)	Initial Setting Time, (min)	Final Setting Time, (min)
C1-4-S-0	21	160	220
C1-4-S-25	19	150	210
C1-4-S-50	17	140	195
C1-4-S-75	14	125	180
C1-5-S-0	25	150	210
C1-5-S-25	23	140	205
C1-5-S-50	22	125	190
C1-5-S-75	20	105	165
C2-4-S-0	18	185	255
C2-4-S-25	17	175	245
C2-4-S-50	15	160	225
C2-4-S-75	13	140	205
C2-5-S-0	20	190	255
C2-5-S-25	19	180	245
C2-5-S-50	18	170	240
C2-5-S-75	16	155	225
C4-4-S-0	19	215	280
C4-4-S-25	18	195	260
C4-4-S-50	17	185	245
C4-4-S-75	15	170	240
C4-5-S-0	21	225	295
C4-5-S-25	19	210	285
C4-5-S-50	18	200	275
C4-5-S-75	16	185	240

**Table 5:** Results of Bingham data fitting of the rheological data of SAPCPs.

Notation	Cement Type	SAP, %	Plastic Viscosity, Pa.s (η)	$\mathrm{Yield}\;\mathrm{Stress},\;\mathrm{Pa}\;(\tau_{0}$)	R
C1-4-S0	CEM I	0	10.443	15.111	0.998
C1-4-S25		0.25	6.952	74.79	0.997
C1-4-S50		0.5	9.818	206.1	0.987
C1-4-S75		0.75	−28.706	1414.8	0.930
C1-5-S0		0	4.929	11.947	0.997
C1-5-S-25		0.25	4.3437	53.978	0.996
C1-5-S50		0.5	5.064	58.338	0.996
C1-5-S75		0.75	−40.819	1438.3	0.933
C2-4-S0	CEM II	0	11.007	24.749	0.998
C2-4-S25		0.25	11.164	66.601	0.998
C2-4-S50		0.5	11.142	135.13	0.996
C2-4-S75		0.75	−36.625	2585.4	0.961
C2-5-S0		0	5.709	13.561	0.989
C2-5-S-25		0.25	5.178	93.448	0.976
C2-5-S50		0.5	5.812	116.45	0.963
C2-5-S75		0.75	−65.747	2199	0.919
C4-4-S0	CEM IV	0	11.016	16.523	0.998
C4-4-S25		0.25	10.98	87.113	0.998
C4-4-S50		0.5	8.59	119.46	0.991
C4-4-S75		0.75	0	2750	N/A *
C4-5-S0		0	6.914	13.643	0.996
C4-5-S-25		0.25	−5.073	379.79	0.690
C4-5-S50		0.5	−64.85	2172.4	0.929
C4-5-S75		0.75	−86.667	4077.5	0.994

* No data can be calculated.

## Data Availability

The data presented in this study are available on request from the corresponding author.
